# Nuclease-mediated gene editing by homologous recombination of the human globin locus

**DOI:** 10.1093/nar/gkt947

**Published:** 2013-10-23

**Authors:** Richard A. Voit, Ayal Hendel, Shondra M. Pruett-Miller, Matthew H. Porteus

**Affiliations:** ^1^Department of Pediatrics, Stanford University, 1291 Welch Rd. Stanford, CA 94305, USA and ^2^Department of Pediatrics, University of Texas Southwestern Medical Center, 5323 Harry Hines Blvd. Dallas, TX 75390, USA

## Abstract

Tal-effector nucleases (TALENs) are engineered proteins that can stimulate precise genome editing through specific DNA double-strand breaks. Sickle cell disease and β-thalassemia are common genetic disorders caused by mutations in β-globin, and we engineered a pair of highly active TALENs that induce modification of 54% of human β-globin alleles near the site of the sickle mutation. These TALENS stimulate targeted integration of therapeutic, full-length beta-globin cDNA to the endogenous β-globin locus in 19% of cells prior to selection as quantified by single molecule real-time sequencing. We also developed highly active TALENs to human γ-globin, a pharmacologic target in sickle cell disease therapy. Using the β-globin and γ-globin TALENs, we generated cell lines that express GFP under the control of the endogenous β-globin promoter and tdTomato under the control of the endogenous γ-globin promoter. With these fluorescent reporter cell lines, we screened a library of small molecule compounds for their differential effect on the transcriptional activity of the endogenous β- and γ-globin genes and identified several that preferentially upregulate γ-globin expression.

## INTRODUCTION

Sickle cell disease is the most common monogenic disease worldwide and is caused by a single point mutation in the β-globin gene. Painful clinical symptoms begin shortly after birth as mutated β-globin subunits replace non-defective γ-globin chains in the predominant form of hemoglobin. Current pharmacological treatment with hydroxyurea partially reverses this globin switching by increasing the production of γ-globin (1,2). This has led to broad interest in developing other compounds and discovering new mechanisms that preferentially upregulate γ-globin (2–5), and also in developing methods to study globin regulation (6,7). Analyses of differential expression of β- and γ-globin generally have been limited to hemoglobin electrophoresis or qRT-PCR, but recent reports have described a method of using the expression of fluorescent molecules driven by the β- and γ-globin promoters as a readout of differential globin regulation. In those studies, the authors integrated into the genome a bacterial artificial chromosome containing the entire 200 kb β-globin locus (which includes both β-globin and γ-globin among other genes), modified such that the β- and γ-globin promoters drive expression of fluorescent proteins (6,7). The integration of the complete genomic locus presumably maintains much of the physiologically relevant regulation of expression, but it does not allow for the direct analysis of the endogenous locus and is confounded by the fact that integration is in a random genomic location and that some cells gain multiple copies of the BAC. In addition, a BAC-based strategy creates a system in which the globin locus is triploid rather than diploid and this change may also affect the regulatory dynamics. Alternatively, direct modification of the endogenous β- and γ-globin loci eliminates those confounding variables.

Endogenous genomic loci can be precisely altered using engineered zinc finger nucleases (ZFNs) (8–11) and Tal-effector nucleases (TALENs) (12–14). ZFNs and TALENs are comprised of a specifically engineered DNA binding domain fused to the FokI endonuclease domain. Binding of a pair of ZFNs or TALENs to contiguous sites leads to the dimerization of the FokI domain, resulting in a targeted DNA double-strand break. Repair of the break can proceed by mutagenic non-homologous end joining or by high-fidelity homologous recombination with a homologous DNA donor template. Compared to ZFNs, TALENs seem to cause lower levels of cytotoxicity (15). Their recognition domain is characterized by repeated arrays of 34 conserved amino acids, except in positions 12 and 13. These two amino acids comprise the repeat variable domain (RVD), which contacts the DNA and provides the nucleotide recognition specificity of each repeat array (16,17). Unlike the other DNA bases which each show strong preference for a single RVD, guanine can be recognized by at least two RVDs with different binding characteristics. The asparagine–asparagine (NN) RVD can form a high-affinity hydrogen bond with guanine, but is not specific because it can also hydrogen bond with adenine (18,19). Conversely, the asparagine-lysine (NK) RVD seems to be more specific for guanine (13) but is less commonly found in naturally occurring TAL-effector proteins (17).

Recent reports have described the development and use of β-globin ZFNs to correct the sickle mutation in human iPS cells. The low rates of confirmed targeting described in these studies (1/300 (20) and 28/286 (21) drug resistant clones were targeted) could be increased by improving the efficiency and toxicity profile of the engineered nucleases. Here, we used highly active and minimally toxic β-globin TALENs to stimulate homologous recombination of therapeutic β-globin cDNA to the endogenous β-globin locus in 19% of cells prior to selection. To analyse the efficiency of both the cutting by the TALENs and the rate of targeted integration, we employed a rapid, accurate and economical deep sequencing method known as single molecule real time (SMRT) sequencing (22). We then describe a new method to generate reporter cells that express fluorescent proteins from endogenous genomic promoters. By using TALENs to target a promoterless GFP in-frame to the endogenous β-globin ATG start site and a promoterless tdTomato in-frame to the endogenous γ-globin ATG start site, we generated a robust endogenous reporter in the context of a common genetic disease. Finally, because γ-globin upregulation is therapeutic in sickle cell disease, we used these fluorescent reporter cells to screen small molecule compounds that preferentially upregulate γ-globin expression compared to β-globin.

## MATERIALS AND METHODS

### Cell lines and transfections

K562 cells (ATCC) were maintained in RPMI 1640 (Hyclone) supplemented with 10% bovine growth serum, 100 units/ml penicillin, 100 µg/ml streptomycin and 2 mM L-glutamine. K562s were transfected by nucleofection (Lonza) using program T-016 and a nucleofection buffer containing 100 mM KH_2_PO_4_, 15 mM NaHCO_3_, 12 mM MgCl_2_ • 6 H_2_0, 8 mM ATP, 2 mM glucose, pH 7.4. HEK293T cells were maintained in DMEM (Cellgro) supplemented with 10% bovine growth serum, 100 units/ml penicillin, 100 µg/ml streptomycin and 2 mM L-glutamine. HEK293T cells were transfected either by calcium phosphate or Lipofectamine 2000 (Invitrogen).

### Nuclease and targeting vector construction

β-globin NK TALENs were synthesized (Genscript) using the Δ152 N-terminal domain and the +63 C terminal domain previously described (13) and fused to the FokI nuclease domain and cloned into pcDNA3.1 (Invitrogen). β-globin NN TALENs and γ-globin NN TALENs were synthesized using a Golden Gate cloning strategy (23) and cloned with the same N- and C- termini and nuclease domain into pcDNA3.1. The β-globin ZFNs were synthesized using the B2H selection strategy previously described (24). The β-Ubc-GFP targeting vector was synthesized by PCR amplifying arms of homology from genomic DNA isolated from K562 cells using the primers in Supplementary Figure S10 and cloning a Ubc-GFP expression cassette in between the arms. The β-in-frame-cDNA and β-in-frame-GFP targeting vectors were synthesized by overlap PCR to insert β-globin cDNA (OriGene) or GFP directly in-frame to the β-globin ATG start codon using the primers in Supplementary Figure S10. Silent mutations were introduced into the β-globin cDNA sequence at every sixth base pair between the nuclease cut site and the end of exon 1. The MGMT P140K drug selection cassette (generous gift from Dr Stan Gerson) was cloned into the targeting vector inside the arms of homology. The γ-in-frame-tdTomato targeting vector was generated by genomic PCR of the 5′ and 3′ arms of homology using primers in Supplementary Figure S10. TdTomato was fused in-frame to the γ-globin ATG start codon by overlap PCR. A neomycin phosphotransferase cassette was cloned in between the arms of homology.

### *In vitro* transcription of nucleases

TALEN and ZFN mRNA was synthesized *in vitro* with the MEGAscript T7 kit (Ambion), polyadenylated *in vitro* with the poly(A) tailing kit (Ambion) and purified with the MEGAclear kit (Ambion) following the manufacturer’s protocols. Two versions of mRNA were synthesized, using unmodified nucleotides or using pseudouridine-5′-triphosphate (Trilink) in place of UTP (25).

### SSA and toxicity assays

A single-strand annealing (SSA) reporter was generated by disrupting the GFP gene by duplicating an internal 42 bp region and separating the duplicated region with a 72 bp fragment from the β-globin region containing the nuclease recognition sites. The SSA reporter and each nuclease were transfected by calcium phosphate into HEK293T cells and analysed on an Accuri C6 flow cytometer (Accuri) after 2 days. The toxicity assay was performed as previously described (24). Briefly, HEK293T cells were co-transfected by calcium phosphate with a pair of nucleases and a GFP expression plasmid. The cells were analysed by FACS for percent GFP positive on day 2 and day 6. The day 2/day 6 ratio was normalized to a non-toxic nuclease sample.

### Surveyor nuclease assay

The Surveyor nuclease assay was performed as previously described (26). Briefly, 6 × 10^5^ HEK293T cells were lipofected with 1.5 µg of each nuclease or 10^6^ K562s were nucleofected with 2.5 µg of each nuclease unless otherwise indicated. After 3 days genomic DNA was isolated using the DNeasy kit (Qiagen) and the locus of interest was PCR amplified using the primers in Supplementary Figure S10 using Accuprime polymerase (Invitrogen). 200 ng of the PCR product was treated with the Surveyor nuclease (Transgenomic) following the manufacturer’s protocol. HEK293T cells were used to characterize the β-globin nucleases because of the presence of a naturally occurring SNP in K562s.

### SMRT sequencing and cDNA targeting

PCR products prior to cutting by the Surveyor nuclease were prepared for SMRT sequencing following the manufacturer’s protocol (Pacific Biosciences). For the SMRT sequencing of the β-globin cDNA targeting events, 10^6^ K562s were nucleofected with 10 µg β-in-frame-cDNA and 1 µg each of βL4 and βR4 TALENs. Aliquots were removed after 3 days when the first round of selection was begun by adding 50 µM O6BG (Sigma) for 1 hour and then adding 40 µM BCNU (Sigma) for 1 hour before changing the media. Cells were allowed to recover for 7–10 days at which time another aliquot was harvested and another round of selection started. Genomic DNA was isolated (Qiagen) and the β-globin region was PCR amplified using primers in Supplementary Figure S10, which did not amplify random integrants. Primers with unique 3 bp tags were used in the PCR reactions from each time point, such that the samples could be combined and analysed in one SMRT sequencing reaction. Data were analysed using CLC Genomics Workbench software.

### Generation of fluorescent reporter cell lines

10^6^ K562 cells were nucleofected with 10 µg of the targeting vector and 1 µg of each TALEN. β-globin-GFP cells were enriched by four rounds of selection with O6BG and BCNU and clones were established by limiting dilution. γ-globin-tdTomato cells were enriched by treatment with 500 µg/ml G418 and clones were established by limiting dilution. Targeting was confirmed by genomic PCR spanning the integration junctions using primers in Supplementary Figure S10.

### Quantitative real-time PCR

Clonal populations of β-globin-GFP cells and γ-globin-tdTomato cells which were targeted at one allele were treated for 4 days with 400 µM hydroxyurea and total mRNA was harvested by Trizol/chloroform extraction and purified on RNeasy columns (Qiagen). 1 µg total RNA was used to synthesize cDNA with the iScript cDNA kit (Bio-Rad) following the manufacturer’s protocol. Biological triplicates were each assayed in triplicate by qRT-PCR using SYBR green (Applied Biosystems) on a CFX384 real-time thermocycler (Bio-Rad) using the primers in Supplementary Figure S10 using the following conditions: initial denaturation (3 min at 95°C), 3-step PCR cycle (10 s at 95°C, 30 s at 55°C, 5 s at 65°C, 40 cycles). PCR efficiency (between 91% and 119%) was calculated using serial dilutions of template for each primer set. mRNA expression was quantified using the 2^−^^ΔΔCt^ method as compared to the housekeeping gene GAPDH.

### Screening globin-modulating compounds

β-globin-GFP cells and γ-globin-tdTomato cells were treated for 4 days with the indicated concentrations of GTP (Sigma), GDP (Sigma), GMP (Sigma), guanosine (Sigma), guanine (Sigma), cGMP (Sigma), Decitabine (Sigma), Sodium butyrate (Sigma), hydroxyurea (Sigma), zileuton (Sigma), hemin (Sigma), cisplatin (Santa Cruz Biotech), pomalidomide (Sigma), mithramycin (Fisher), apicidin (Sigma), cytarabine (Sigma) or phenylacetate (Sigma). Fluorescence was measured using an Accuri C6 cytometer (Accuri), and was reported as the fold change in fluorescence intensity after 4 days.

### Statistical analysis

Data from at least three samples were used to determine significance by statistical analysis. Mean ± SD is reported. Statistical significance was determined by Student’s *t*-test and *P*-values < 0.05 were considered significant.

## RESULTS

### Design and characterization of β- and γ-globin TALENs

**Figure 1.**
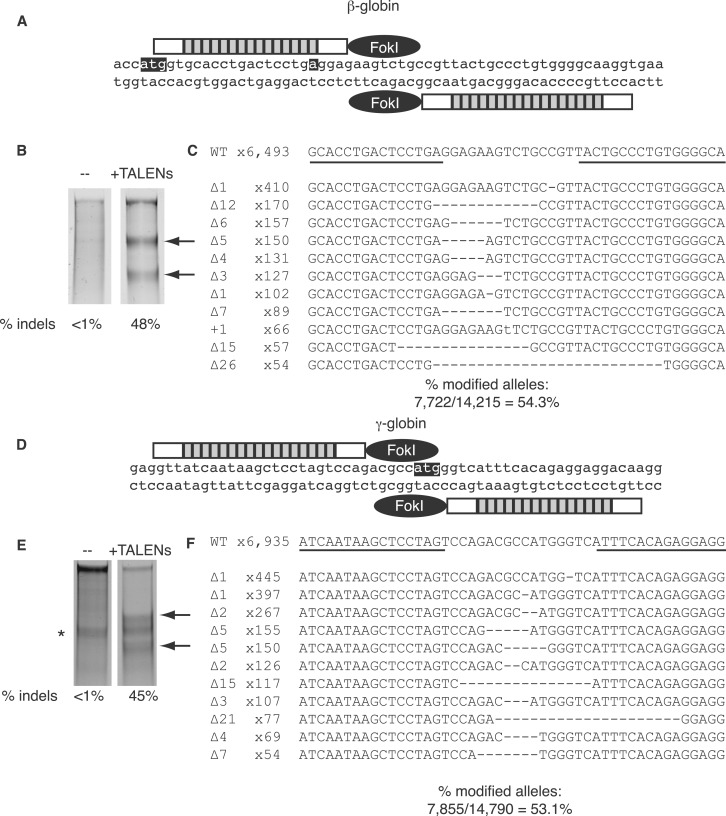
TALEN-mediated disruption at the human β-globin and γ-globin loci. (**A**) Schematic of the βL4/βR4 TALEN binding site in the human β-globin gene. The ATG start codon and the sickle mutation are highlighted. (**B**) β-globin gene disruption in HEK293T cells. Arrows indicate specific Surveyor nuclease cleavage products (**C**) SMRT sequencing of β-globin alleles mutated by treatment with βL4/βR4 TALENs. The 11 most abundant mutated alleles are shown, and the frequency of each is indicated. TALEN binding sites are underlined. (Δ represents deletions, + represents insertions). (**D**) Schematic of the γL3/γR2 TALEN binding site in the human γ-globin gene. The ATG start codon is highlighted. (**E**) γ-globin gene disruption in K562 cells. (*, non-specific cleavage product). (**F**) SMRT sequencing of γ-globin alleles mutated by treatment with γL3/γR2 TALENs. The 11 most abundant mutated alleles are shown, and the frequency of each is indicated. TALEN binding sites are underlined.

To develop a system that robustly and rapidly reports on the activity of both the β-globin and γ-globin loci, we designed a gene-targeting strategy using engineered nucleases. Recent reports have described low but significant levels of genome modification at the endogenous β-globin locus using ZFNs (20,21), and we first sought to improve the rate of gene targeting at the β-globin locus by designing custom TALENs to that site. First, we identified four putative left (βL1-βL4) and four right (βR1-βR4) TALEN binding sites near the sickle mutation in β-globin (Figure 1A and Supplementary Figure S1), and synthesized the eight individual TALENs using the NK RVD to bind each guanine. Notably, we made slight modifications of the final TALEN expression vector to include the N- and C-terminal TALEN truncations that have been shown to be sufficient for optimal TALEN activity (13). In an extrachromosomal SSA assay, we identified six TALEN pairs that stimulated SSA at least 10-fold above background (Supplementary Figure S2). We then re-constructed the most active TALEN pair (βL4-NK/βR4-NK) to contain the NN RVD (βL4-NN/βR4-NN) using the Golden Gate cloning strategy previously described (23). To investigate their activities at the endogenous chromosomal β-globin locus, we used the Surveyor nuclease assay in HEK293T cells. 293T cells were used instead of hematopoietic K562 cells because a SNP in one β-globin allele of K562s confounded analysis in the Surveyor nuclease assay (data not shown). The NK versions modified up to 18% of alleles (Supplementary Figure S5A) and the NN TALENs modified 48% of alleles (Figure 1B and Supplementary Figure S5A). As a comparison, we also used a modification of the ‘oligomerized pool engineering’ (OPEN) method to generate ZFNs to the β-globin locus (24,27). These ZFNs were made independently from the ones reported by Sebastiano *et al.* (21) but are designed to the same target sequence and are very similar in the amino acid sequence of the alpha-helices that mediate DNA binding (Supplementary Figure S3). Although the ZFNs were much more cytotoxic than were the TALENs (Supplementary Figure S4), the ZFNs were also active, modifying up to 12% of β-globin alleles in the Survyeor nuclease assay (Supplementary Figure S5A). Interestingly, delivery of TALENs as mRNA did not increase the already high frequency of cutting, but delivery of the ZFNs as mRNA increased the signal from 12% to 35% (Supplementary Figure S5B). Importantly, the TALENs showed only 4% modification at the δ-globin locus (Supplementary Figure S5C), which has high sequence homology with β-globin (Supplementary Figure S1).

To confirm the frequency of genome modification by βL4-NN/βR4-NN, we used SMRT sequencing, a rapid, high-throughput method for sequencing of the β-globin locus following TALEN treatment (22). SMRT sequencing allows for simultaneous analysis of up to 30 000 sequences, as well as multiplexing various samples at once. Analysis of 14 215 β-globin sequences revealed TALEN modification of 54% (Figure 1C).

Next, to modify the endogenous γ-globin locus, we designed and constructed three left (γL1– γL3) and two right (γR2– γR3) NN TALENs that bind sequences near the ATG start codon of γ-globin (Figure 1D and Supplementary Figure S6). Because of the sequence identity between Aγ-globin and Gγ-globin these TALEN pairs do not distinguish the two loci. To measure the activity of the γ-globin TALENs, we again used the Surveyor nuclease assay, which resulted in modification of up to 44% of γ-globin alleles with the γL3/γR2 pair (Figure 1E). Two other TALEN pairs modified >30% of γ-globin alleles (Supplementary Figure S7). Analysis of 14 790 γ-globin SMRT sequences revealed a modification rate of 53% with γL3/γR2 (Figure 1F). As expected, because of the lack of sequence homology between the β-globin and γ-globin loci, the γ-globin TALENs had no activity at the β-globin locus (data not shown).

### TALEN-mediated β-globin targeting by homologous recombination

**Figure 2.**
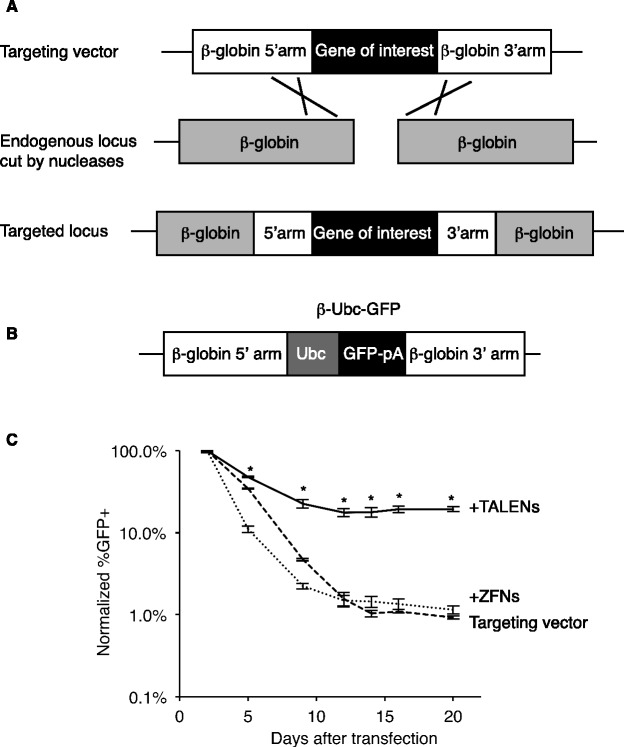
High-frequency gene targeting using β-globin TALENs. (**A**) Schematic of nuclease-mediated gene targeting to the endogenous β-globin locus. (**B**) β-Ubc-GFP targeting vector. Ubc, ubiquitin C promoter; pA, BGH polyadenylation signal sequence. (**C**) Gene targeting of β-Ubc-GFP to the endogenous β-globin locus in K562 cells using βL4/βR4 TALENs and ZFNs (*P < 0.005 compared to targeting vector alone). Each experimental condition was performed in biological triplicate.

We then sought to determine at what frequency these highly active TALENs stimulated gene targeting by homologous recombination (Figure 2A). First to target the β-globin locus, we designed a targeting vector with ∼1 kb arms of homology 5′ and 3′ of the TALEN cut site. In between the homology arms, we included a Ubc-GFP expression cassette that, upon successful homologous recombination, would be stably integrated into the β-globin locus (Figure 2B, ‘β-Ubc-GFP’ targeting vector). Gene targeting was achieved by nucleofection of β-Ubc-GFP with βL4-NN and βR4-NN TALEN expression plasmids into erthyroleukemic K562 cells, and resulted in stable integration of Ubc-GFP in 19% of transfected cells (13% overall) compared to <1% in the absence of TALENs (Figure 2C). We then compared the activities of the NK and NN β-globin TALENs in the gene-targeting assay. In confirmation of the Surveyor assay data, the NN versions stimulated a significantly higher rate of targeted integration compared to the NK TALENs. Interestingly, when paired with βR4-NN, both βL4-NK and βL4-NN stimulate high rates of targeting (∼20%). However, when paired with βR4-NK, βL4-NK resulted in 1.8% stable GFP expression, while βL4-NN led to 4.5% stable GFP expression (Supplementary Figure S8). Despite high rates of modification in the Surveyor assay (Supplementary Figure S5A), the ZFNs did not stimulate targeting of the β-globin locus and targeted integration of the Ubc-GFP cassette could not be discriminated from background random integrants (Figure 2C and Supplementary Figure S8). In this direct comparison of ZFNs and TALENs designed to target nearly the same sequence (Supplementary Figure S1), we found that the TALENs were significantly better because of their greater cutting activity, significantly greater stimulation of targeting and their lower toxicity. These data also demonstrate better activity with TALENs using NN as the RVD to recognize guanine compared to NK but that NK TALENs can have excellent activity in the correct context.

### Targeting β-globin cDNA to the endogenous β-globin locus

**Figure 3.**
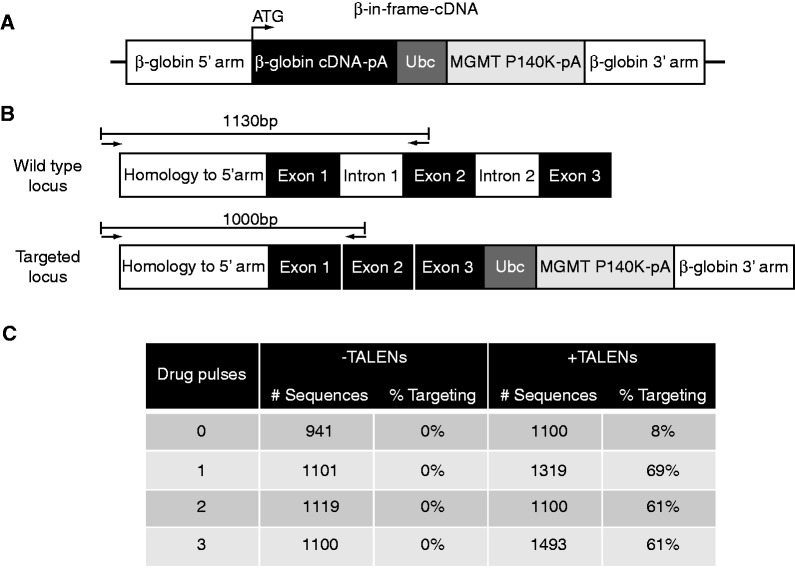
Targeting therapeutic β-globin cDNA to the endogenous β-globin locus. (**A**) β-in-frame-cDNA targeting vector. When targeted, the cDNA is expressed from the endogenous ATG start site. pA, BGH polyadenylation signal sequence; Ubc, Ubiquitin C promoter; MGMT, Methylguanine methyltransferase. (**B**) Schematic of the wt (top) and targeted (bottom) β-globin locus. The absence of intron 1 in the cDNA-targeted locus results in a shorter PCR product. (**C**) Results from SMRT sequencing of targeted β-globin alleles without selection (top row) and with up to three rounds of drug selection.

We next sought to target full-length β-globin cDNA to the endogenous β-globin ATG start site. In this way, endogenous β-globin regulatory elements would express β-globin from the cDNA instead of from the wild-type genomic sequence, a strategy that would be clinically relevant for both sickle cell disease and β-thalassemia. We modified the β-Ubc-GFP targeting vector, replacing the Ubc-GFP cassette with β-globin cDNA fused in-frame to the natural β-globin ATG start codon, already present in the 5′ arm of homology (Figure 3A, ‘β-in-frame-cDNA’ targeting vector). Also included in the β-in-frame-cDNA targeting vector was a drug selection cassette encoding a mutant form of methylguanine methyltransferase (MGMT P140K), which allowed for enrichment of targeted cells by treatment with the combination of O6-benzylguanine (O6BG) and carmustine (BCNU).

To determine the frequency of targeting and the efficiency of drug selection, we again employed SMRT sequencing. First, we targeted K562s with the β-in-frame-cDNA targeting vector using βL4/βR4 TALENs. Then we pulsed the samples three times with O6BG and BCNU and harvested gDNA after each pulse. To amplify the β-globin locus, we used a forward primer that is 5′ and outside the start of the 5′ homology arm and a reverse primer in exon 2 of β-globin (Figure 3B). In this way, random integrants were not amplified. The presence of intron 1 in the wild-type genomic DNA sequence of this locus, and its absence in the targeted β-globin cDNA, allowed us to determine the ratio of targeted alleles to wild-type alleles after each pulse based on the length of the sequence, which could then be confirmed by the sequence content (Figure 3B). In the absence of drug selection, 8% of the alleles were targeted as determined by analysing the sequence of 1100 alleles in the TALEN-treated sample. The targeting frequency of 8% of alleles is consistent with the observed rate of β-Ubc-GFP targeting in 19% of cells because there are three copies of the β-globin locus in K562 cells (Figure 2C). Pulsing the targeted cells with O6BG/BCNU up to three times resulted in the enrichment of targeted alleles such that they accounted for >60% of all sequenced alleles (Figure 3C). Since K562s are known to be aneuploid with three copies of the globin locus (28), a post-selection modified allele frequency of 60% is consistent with a highly purified population in which nearly 100% of cells are targeted at one or multiple β-globin alleles.

### Generation of fluorescent β- and γ-globin reporters by endogenous locus tagging

**Figure 4.**
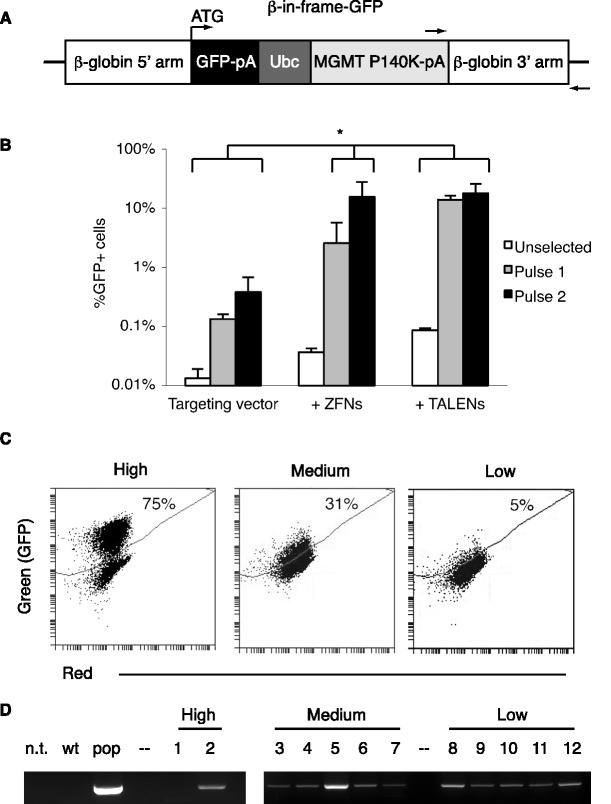
Generation of β-globin reporter cells by in-frame GFP targeting to the endogenous β-globin locus. (**A**) β-in-frame-GFP targeting vector. When targeted, GFP is expressed from the endogenous ATG start site. Arrows indicate PCR primers used in Panel D to confirm targeting. pA, BGH polyadenylation signal sequence; Ubc, Ubiquitin C promoter; MGMT, Methylguanine methyltransferase. (**B**) Targeting of β-in-frame-GFP to the endogenous β-globin locus using βL4/βR4 TALENs and ZFNs (*P < 0.05 compared to targeting vector alone). Each experimental condition was performed in biological triplicate. (**C**) FACS plots of targeted clones, classified by level of GFP expression. (**D**) Genomic PCR using primers in Panel A to detect presence of a targeted β-globin locus in 11 of 12 GFP positive clones. n.t., no template control; wt, genomic DNA from wild-type cells; pop, genomic PCR from targeted population; –, no sample.

Next, we redesigned the β-Ubc-GFP targeting vector such that a promoterless GFP was fused in-frame to the β-globin ATG start codon (Figure 4A, ‘β-in-frame-GFP’ targeting vector). In this way, upon targeting to the endogenous β-globin locus, GFP would be driven by the endogenous β-globin promoter and would be subject to the regulatory elements controlling β-globin expression. We targeted the β-in-frame-GFP targeting vector to the β-globin locus, using either βL4/βR4 TALENs or ZFNs. Under the same experimental conditions that resulted in targeting rates of 19% with the β-Ubc-GFP targeting vector, the β-in-frame-GFP targeting experiment resulted in a much lower percentage of GFP positive cells, which is attributable not to lower targeting frequencies but to the naturally low level of β-globin expression in K562s (28,29). That is, the level of GFP expression driven by the β-globin gene is too low to be seen above background in many cells. Nonetheless, in the presence of βL4 and βR4 TALENs, there was a significantly higher percentage of GFP positive cells than in control samples (Figure 4B, white bars). Selection with two pulses of O6BG and BCNU resulted in significant enrichment of GFP positive cells in the TALEN and ZFN samples compared to the targeting vector alone (Figure 4B). Notably, with up to four pulses with O6BG and BCNU, the overall percentage of GFP positive cells never increased above 20% (data not shown). We believe this is due to the low activity of the β-globin promoter in K562s. When we sorted for GFP positive cells from the TALEN sample, over the course of 2 weeks in culture, the population went from being >95% GFP positive to ∼15% (data not shown). A second sort again resulted in a population of >95% GFP positive cells that fell to 15%. We attributed this observation to the low level of β-globin expression in K562s, such that at any given time 15% of the population expressed GFP at a high enough level to be detected by flow cytometry. When we analysed 48 individual clones from the drug selected TALEN sample, we observed three distinct patterns of GFP expression that we designated ‘high,’ ‘medium’ and ‘low’ (Figure 4C). To determine whether these clonal populations expressed GFP because of targeting to the β-globin locus, we used a genomic PCR assay spanning the junction of integration (Figure 4A, arrows). In this way, the presence of a PCR product indicates correct targeting to the endogenous β-globin locus. Indeed, 11 of 12 analysed clones showed targeted integration (Figure 4D). Interestingly, the one clone that did not produce a PCR product and thus was not targeted (clone #1) was a ‘high’ GFP expressing clone that had undergone random integration. Although we did not investigate the specific site of integration in this clone, based on its expression profile, it was likely near strong promoter elements that drive robust expression of the transgene. Of the original 48 clones, only 4 had ‘high’ GFP expression, corresponding to the absence of targeting to the β-globin locus by junction PCR (data not shown). These data demonstrate that targeted cells show low levels of GFP expression because of the low activity of the β-globin promoter in K562s, and that high-expressing cells are paradoxically more likely to be the result of random integration.

**Figure 5.**
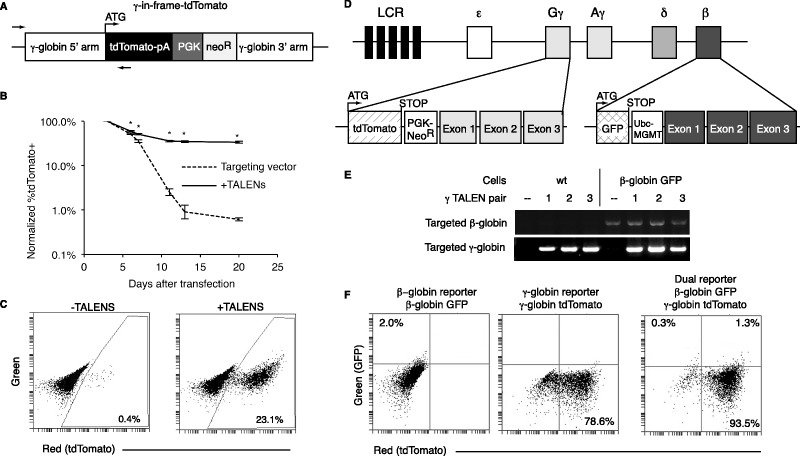
Generation of fluorescent γ-globin and dual-globin reporter cell lines. (**A**) γ-in-frame-tdTomato targeting vector. When targeted, tdTomato is expressed from the endogenous ATG start site. Arrows indicate PCR primers used in Panel E to confirm targeting. pA, BGH polyadenylation signal sequence; PGK, Phosphoglycerate kinase promoter; neo^R^, Neomycin phosphotransferase. (**B**) Targeting of γ-in-frame-tdTomato to the endogenous γ-globin locus using γL3/γR2 TALENs (*P < 0.005 compared to targeting vector alone). Each experimental condition was performed in biological triplicate. (**C**) FACS plots showing stable integration of tdTomato on day 20 in the presence of TALENs. (**D**) Schematic of the targeted globin loci showing tdTomato being expressed from the endogenous γ-globin ATG start site and GFP from the endogenous β-globin ATG start site. The wild-type γ- and β-globin gene sequences are still present after targeting but are not expressed because of the stop codons that follow the targeted tdTomato and GFP sequences. (LCR, locus control region). (**E**) Genomic PCR using primers in Panel A to detect presence of a targeted γ-globin locus in samples treated with γ-globin TALENs (Pair 1: γL3/γR2, pair 2: γL3/γR3, pair 3: γL2/γR2). Wild-type cells (left) were targeted to generate the γ-globin tdTomato reporter line, and β-globin-GFP cells (right) were targeted to generate the dual reporter cell line. (**F**) FACS plots showing the fluorescent profile of the β-globin-GFP reporter (left) the γ-globin-tdTomato reporter (center) and β-globin-GFP/γ-globin-tdTomato dual reporter (right).

To develop a fluorescence-based reporter of the endogenous γ-globin locus, we targeted tdTomato in-frame to the ATG start codon of γ-globin, using a homologous targeting vector containing in-frame tdTomato followed by a neomycin drug resistance cassette (Figure 5A, ‘γ-in-frame-tdTomato’). Unlike β-globin, γ-globin is highly expressed in K562 cells so the fluorescent readout from the targeted γ-in-frame-tdTomato accurately reflected the overall integration rate despite the lack of an exogenous promoter. Co-transfection of γ-in-frame-tdTomato with γL3/γR2 TALENs resulted in stable tdTomato expression in 34% of transfected cells (23% overall), compared to <1% in samples without TALENs (Figure 5B and C). Genomic PCR spanning the integration junction (Figure 5A, arrows) revealed the presence of a targeted band in samples treated with any of the three most active pairs of γ-globin TALENs (Figure 5E, left).

To create a dual-fluorescent reporter that expresses GFP from the endogenous β-globin locus and tdTomato from the endogenous γ-globin locus (Figure 5D), we used the γ-globin TALENs to target the γ-in-frame-tdTomato vector to the γ-globin locus in a previously targeted β-globin-GFP clone (Figure 5E, right). In this way, we generated three cell lines that report on the activity of endogenous globin promoters, the β-globin-GFP reporter, γ-globin-tdTomato reporter and the β-globin-GFP/ γ-globin-tdTomato dual reporter (Figure 5F). In the clones selected as reporter cell lines, expression from the fluorescent transgenes remained stable over the course of more than 4 months in culture.

### Using endogenous fluorescent reporter cells to screen globin-modulating compounds

**Figure 6.**
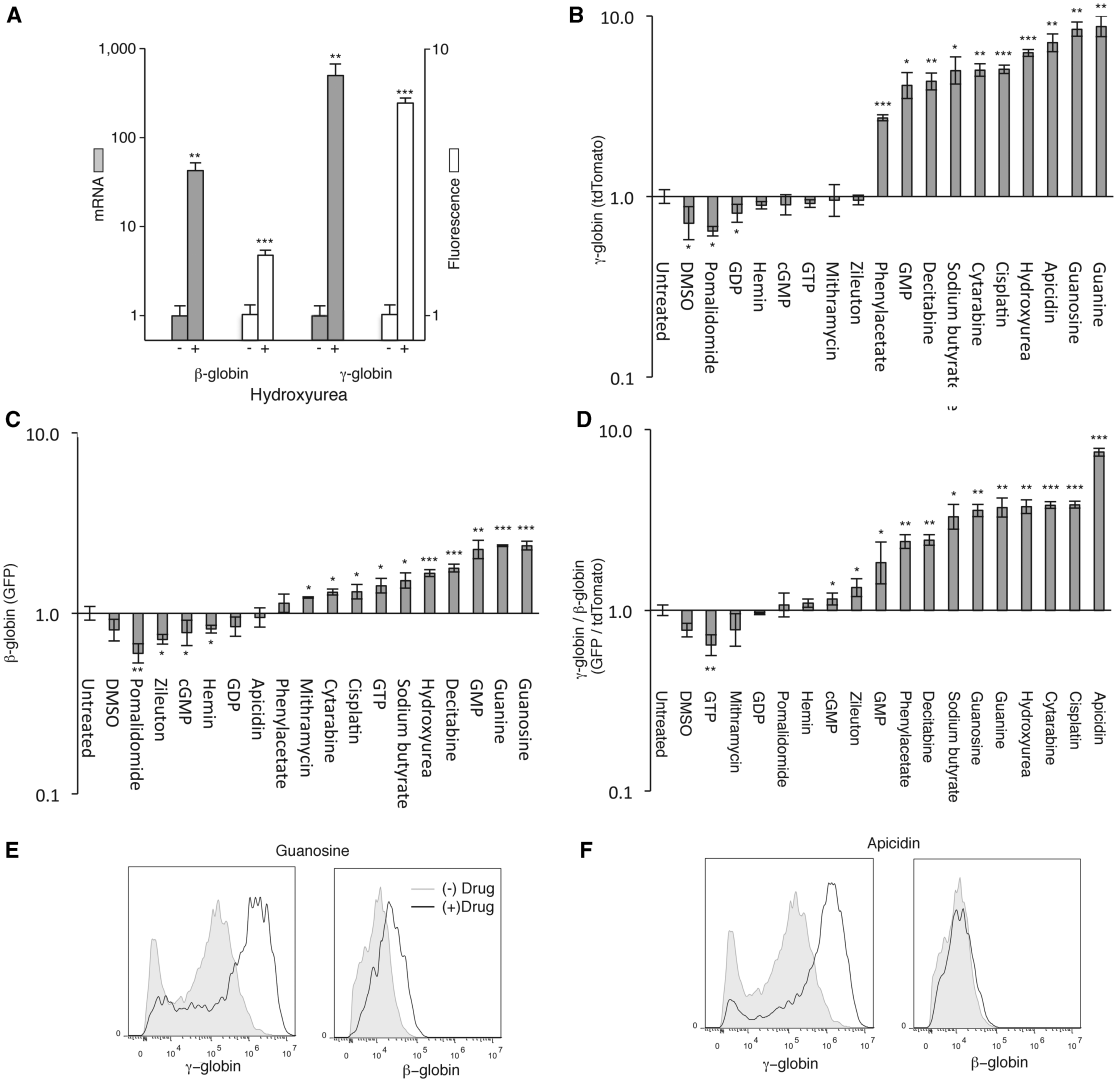
Using fluorescent reporter cell lines to screen globin-modulating compounds. (**A**) Effect of 400 µM hydroxyurea on β- and γ-globin transcript levels (gray bars) and on GFP and tdTomato expression in targeted cell lines (white bars). Effect of drug treatment on (**B**) tdTomato expression in γ-globin tdTomato cells, (**C**) GFP expression in β-globin-GFP cells and (**D**) the ratio of tdTomato/GFP expression in γ-globin-tdTomato and β-globin-GFP cells. FACS plots showing the effect on the expression of tdTomato and GFP from the γ-globin and β-globin loci of (**E**) guanosine, and (**F**) apicidin. *Drug concentrations*: 0.2% DMSO, 10 µM pomalidomide, 400 µM GDP, 5 µM hemin, 400 µM cGMP, 200 µM GTP, 200 nM mithramycin, 200 µM zileuton, 4 mM phenylacetate, 400 µM GMP, 10 µM decitabine, 1200 µM sodium butyrate, 5 µM cytarabine, 4 µM cisplatin, 400 µM hydroxyurea, 400 nM apicidin, 400 µM guanosine and 400 µM guanine.

Next, we sought to establish these fluorescent reporter lines as tools that can be used to compare the globin-modulating activities of small molecule compounds. Hydroxyurea, is used clinically to increase the production of γ-globin and it has been shown to upregulate γ-globin in K562s (30,31). K562s treated for 4 days with 400 µM hydroxyurea showed a significant 62-fold increase in β-globin expression as measured by qRT-PCR. γ-globin mRNA levels were even more elevated than β-globin transcripts after treatment with hydroxyurea, increasing 932-fold (Figure 6A). Next, we treated the β-globin-GFP reporter cells and the γ-globin-tdTomato reporter cells with hydroxyurea and measured mean fluorescence intensity on day 4. GFP and tdTomato intensities were significantly higher compared to untreated cells, and the increase in tdTomato was significantly greater than the increase in GFP, mirroring the changes in β- and γ-globin expression levels. These results show that the reporter cell lines can be used to rapidly, accurately and robustly measure the activity of the endogenous globin loci.

To expand our analysis, we treated cells from the β-globin-GFP, γ-globin-tdTomato and β-globin-GFP/γ-globin-tdTomato cell lines with 5 concentrations of 17 different compounds shown previously to modulate globin expression (Supplementary Figure S9). Of these, 10 significantly increased the expression of the endogenous γ-globin locus, the most striking of which were guanine, guanosine, apicidin and hydroxyurea (Figure 6B). Similarly 10 compounds increased the expression of endogenous β-globin, with the best inducers being guanosine, guanine and GMP (Figure 6C). The ideal pharmacological therapy for sickle cell disease is a drug that preferentially induces the production of γ-globin compared to β-globin. Therefore the most relevant analysis was of the ratio of induction of γ- to β-globin (Figure 6D). Compounds such as guanosine increased the expression of both γ- and β-globin (Figure 6E). However, apicidin was a strong inducer of γ-globin but had no activity at the β-globin promoter (Figure 6F). Importantly hydroxyurea, the clinical standard of care for induction of γ-globin had one of the highest γ/β induction ratios of all the screened compounds. In this way, we have established a system to robustly, rapidly and simultaneously report on the activity of the endogenous β- and γ-globin promoters.

## DISCUSSION

The emergence of the TALEN platform for engineering nucleases has made possible the rapid, open-source generation of highly active genome editing proteins. TALENs have been used to cause site-specific gene disruption and gene targeting in yeast (32,33), plants (23), nematodes (34), zebrafish (35,36), rats (37) and human cells (12–14). A recent report described TALENs designed to human β-globin and showed 5% gene correction of a mutated GFP gene, which had been disrupted by the insertion of the β-globin sequence recognized by the TALENs, but did not describe their activity at the endogenous β-globin locus (38). The authors then the used β-globin TALENs and a transposon-based targeting strategy to correct the sickle mutation in patient-derived iPS cells (39). In a third report, Cradick *et al.* designed a CRISPR/Cas9 system to target β-globin and showed efficient modification of the endogenous locus but demonstrated significant off-target effects (40). Here, we synthesized and compared the activities of NN-TALENs, NK-TALENs and ZFNs designed to the same genomic region in the human β-globin gene. We sought to induce a DNA double-strand break near the site of the sickle mutation, which limited the number of potential TALEN binding sites that adhered to the 5′T design rule (23). Although several of the TALENs without a 5′T did show nuclease activity at the β-globin sequence (Supplementary Figure S2), notably the most active pair βL4/βR4 adhered to the 5′T rule. The highly active βL4 TALEN monomer was designed such that the most C-terminal RVD binds to the sickle thymine and not the wild-type adenine. In spite of this 1 bp mismatch, βL4/βR4 were as active as the ‘wild-type sequence’ βL4/βR4 TALENs in non-sickle cell lines (data not shown). The promiscuity of the TALEN pair designed to the sickle site for the wild-type sequence highlights the necessity of a thorough analysis of off-target effects of this nuclease pair. However, the activity at the wild-type sequence itself is not a concern in the potential therapeutic applications of this TALEN pair, as it would only be clinically used in patients with two mutated alleles.

Using the βL4/βR4 TALENs, we targeted β-globin cDNA to the ATG start codon of the endogenous β-globin locus in human cells and used deep sequencing method to precisely detect rates of targeting. Then we developed a TALEN-based locus tagging method to report on the activity of endogenous promoters by targeting GFP and tdTomato to the start codons of the endogenous β-globin and γ-globin genes, respectively. Finally, we showed that our endogenously tagged reporter cells provide a rapid and facile method to analyse the globin-modulating activities of small molecule compounds.

Our strategy of using SMRT sequencing to validate the activity of engineered nucleases as determined by the Surveyor nuclease assay allows for the analysis of many more sequences as compared to standard Sanger sequencing methods at a fraction of the cost of other deep sequencing platforms such as Illumina. We believe that using deep sequencing to determine cutting and targeting frequencies will be especially beneficial in primary cells such as CD34+ hematopoietic stem cells in which these rates are considerably lower compared to cell lines.

Using TALENs to target full-length β-globin cDNA to the endogenous β-globin locus provides an alternate method to gene conversion of the sickle mutation using ZFNs as recently described (20,21). First, we showed considerably higher nuclease activity, using a TALEN platform that has been shown in side-by-side comparisons to be less toxic than ZFNs (15). In terms of toxicity, we showed using a previously described toxicity assay that the βL4/βR4 TALENs have considerably less cellular toxicity than both the β-globin ZFNs and the widely used CCR5 ZFNs (Supplementary Figure S4). Analysis of the highly similar δ-globin locus revealed that the βL4/βR4 TALENs have minimal activity at that site (Supplementary Figure S5). True genome-wide, site-specific analysis for off-target activity is the focus of ongoing research.

With regard to nuclease activity, in the β-in-frame-GFP targeting experiments that have low background signal because of the lack of exogenous promoter, we could detect targeted integration with the ZFNs after drug selection, showing that the ZFNs are capable of stimulating gene targeting at the β-globin locus. However, we were unable to detect targeting of the β-Ubc-GFP cassette with ZFNs at levels above background random integration, presumably due to extremely low targeting and the toxicity of the ZFNs. In summary, our βL4/βR4 TALENs are more active and less toxic than OPEN-generated ZFNs in both genomic and functional assays.

Another improvement in our strategy is that cDNA targeting would be therapeutic in both sickle cell disease, in which the causative mutation is at codon 6 of the β-globin gene, and β-thalassemia, in which causative mutations can occur throughout the length of the β-globin gene. The co-conversion of the sickle mutation with the downstream integration of a drug resistance cassette in the first intron as described (20,21) has been demonstrated to be less efficient in cases when there is homologous sequence in between the site of the conversion and the insertion of the selectable marker (41) such as the first exon of β-globin. Therefore, when we designed the β-in-frame-cDNA targeting vector we introduced silent mutations in every sixth nucleotide of the cDNA sequence between the nuclease cut site and the end of the first exon. By reducing the homology between the genomic locus and the cDNA, we shunted the repair to proceed via homologous recombination with the 3′ arm of homology (instead of with the short stretch of homology in exon 1 of the cDNA), ensuring that the drug selection cassette is also targeted to the locus. Unlike previous gene therapy trials that relied on random integration of β-globin and described the importance of β-globin introns on the expression of the transgene (42), our next-generation approach directly modifies the endogenous locus preserving the extra-genic regulatory elements. Because the intervening sequence 2 (IVS2) has been shown to increase expression of β-globin cDNAs up to 500-fold (43), if we find that expression of the β-globin cDNA is too low in primary cells, we can test whether adding the IVS2 sequence to the construct to increase expression. In contrast to prior experiments testing the importance of IVS2, in our targeting experiments, IVS2 is retained at the locus and thus any regulatory effects it might have could be still be preserved. The effect that including this intronic sequence in the targeting construct would have on the efficiency of homologous recombination would also have to be tested. Additionally, we chose to use the MGMT P140K-based drug selection strategy because it is effective *in vitro* (44) and relies on the FDA-approved compounds O6BG and BCNU, which can enrich for targeted cells *in vivo* (45).

Dozens of reports have analysed the effect of drugs on globin expression, primarily by analysing transcript levels by qRT-PCR, hemoglobin electrophoresis or benzidine staining (4,6,7,46–55). We established a method to generate fluorescent cell lines as accurate reporters of differential globin expression, and validated them by comparing the induction of β- and γ-globin mRNA transcripts with the increase in GFP and tdTomato signal following treatment with hydroxyurea. Although we did not directly control for potential cell cycle effects on fluorescence alteration following drug treatment, analysis of transcript level by qRT-PCR validates that these changes in fluorescence are due to the modification of gene expression. Then we used the fluorescent globin reporters in a mini drug screen to demonstrate their utility as tools to rapidly and accurately measure modulations in globin expression. Despite using compounds that have been previously described to be γ-globin inducers, we found that more than half of them also significantly increased expression from the β-globin locus. One mechanism by which small molecule compounds affect globin expression is through the induction of erythroid differentiation (29). The degree to which these compounds affected the extent of differentiation of this cell line and the mechanism of globin-induction by these compounds was not directly investigated here. No matter the mechanism of induced globin expression, these data highlight the importance of simultaneously evaluating both β- and γ- globin expression in globin-induction studies. This is the first proof-of-principle example of using precise genome engineering to rapidly and efficiently generate cell lines with endogenous promoter reporters, validating the output by direct comparison to mRNA transcript levels, and then using the dual reporter cell line to screen for small molecules that differentially regulate two genes. In this way, we introduce a novel method to analyse endogenous promoter activity in the context of the most prevalent monogenic disease.

Historically, many globin expression studies were done in K562 cells because they are ubiquitous erythroid precursors and are highly amenable to *in vitro* experimentation. However despite their widespread use, K562s are an imperfect system with which to study the intricacies of globin biology because of the non-physiological levels of β-and γ- globin expression. Indeed, our results similarly show a very high level of baseline tdTomato expression in the γ-in-frame-tdTomato targeted cells with a low basal level of GFP expression from the β-in-frame-GFP cells. Despite this, we are able to demonstrate robust differential expression of β- and γ-globin upon induction by various pharmacological compounds, including high γ/β induction with hydroxyurea, the only compound clinically approved for this purpose. With these limitations in mind, we chose K562s as our model system because they can tolerate transfection of large amounts of DNA, allowing for optimization of the vital genome engineering aspects of this strategy. It is clear that alternative cell lines and ultimately primary erythroid progenitors are required to mechanistically describe and validate the methods of globin modulation that are suggested in this proof-of-principle work. As transfection methods of primary cells improve and with the discovery of potentially less toxic modified RNAs, we anticipate achieving biologically relevant levels of genome modification in these cells.

Despite the limitations of K562s, there have been no fewer than 20 reports in the literature in the last year alone describing globin modulation in this cell line. Here, we describe a novel method to concurrently evaluate β- and γ-globin expression, using compounds that have been previously described to regulate globin expression. Having validated the effectiveness of this multi-fluorescent endogenous globin expression approach, we are now transitioning this work into a more biologically relevant cell line which we can use in an unbiased high-throughput drug screen to identify novel γ-globin-specific inducers to be the next generation of pharmacologic therapy for patients with sickle cell disease. More generally, this strategy could be broadly applied to generate multi-color reporter cell lines to allow rapid screening for conditions and compounds that promote the activity of a particular pathway or determine cellular fate.

## SUPPLEMENTARY DATA

Supplementary Data are available at NAR Online.

Supplementary Data

## References

[1] Lavelle DE (2004). The molecular mechanism of fetal hemoglobin reactivation. Semin. Hematol..

[2] Steinberg MH, Rodgers GP (2001). Pharmacologic modulation of fetal hemoglobin. Medicine (Baltimore).

[3] Bauer DE, Orkin SH (2010). Update on fetal hemoglobin gene regulation in hemoglobinopathies. Curr. Opin. Pediatr..

[4] Liu K, Xing H, Zhang S, Liu S, Fung M (2010). Cucurbitacin D induces fetal hemoglobin synthesis in K562 cells and human hematopoietic progenitors through activation of p38 pathway and stabilization of the gamma-globin mRNA. Blood Cells Mol. Dis..

[5] Xu J, Peng C, Sankaran VG, Shao Z, Esrick EB, Chong BG, Ippolito GC, Fujiwara Y, Ebert BL, Tucker PW (2011). Correction of sickle cell disease in adult mice by interference with fetal hemoglobin silencing. Science.

[6] Chan KS, Xu J, Wardan H, McColl B, Orkin S, Vadolas J (2012). Generation of a genomic reporter assay system for analysis of gamma- and beta-globin gene regulation. FASEB J..

[7] Howden SE, Voullaire L, Wardan H, Williamson R, Vadolas J (2008). Site-specific, Rep-mediated integration of the intact beta-globin locus in the human erythroleukaemic cell line K562. Gene. Ther..

[8] Hockemeyer D, Soldner F, Beard C, Gao Q, Mitalipova M, DeKelver RC, Katibah GE, Amora R, Boydston EA, Zeitler B (2009). Efficient targeting of expressed and silent genes in human ESCs and iPSCs using zinc-finger nucleases. Nat. Biotechnol..

[9] Perez EE, Wang J, Miller JC, Jouvenot Y, Kim KA, Liu O, Wang N, Lee G, Bartsevich VV, Lee YL (2008). Establishment of HIV-1 resistance in CD4+ T cells by genome editing using zinc-finger nucleases. Nat. Biotechnol..

[10] Urnov FD, Miller JC, Lee YL, Beausejour CM, Rock JM, Augustus S, Jamieson AC, Porteus MH, Gregory PD, Holmes MC (2005). Highly efficient endogenous human gene correction using designed zinc-finger nucleases. Nature.

[11] Voit RA, McMahon MA, Sawyer SL, Porteus MH Generation of an HIV resistant T-cell line by targeted “stacking” of restriction factors. Mol. Ther..

[12] Hockemeyer D, Wang H, Kiani S, Lai CS, Gao Q, Cassady JP, Cost GJ, Zhang L, Santiago Y, Miller JC (2011). Genetic engineering of human pluripotent cells using TALE nucleases. Nat. Biotechnol..

[13] Miller JC, Tan S, Qiao G, Barlow KA, Wang J, Xia DF, Meng X, Paschon DE, Leung E, Hinkley SJ (2010). A TALE nuclease architecture for efficient genome editing. Nat. Biotechnol..

[14] Reyon D, Tsai SQ, Khayter C, Foden JA, Sander JD, Joung JK (2012). FLASH assembly of TALENs for high-throughput genome editing. Nat. Biotechnol..

[15] Mussolino C, Morbitzer R, Lutge F, Dannemann N, Lahaye T, Cathomen T (2011). A novel TALE nuclease scaffold enables high genome editing activity in combination with low toxicity. Nucleic Acids Res..

[16] Boch J, Scholze H, Schornack S, Landgraf A, Hahn S, Kay S, Lahaye T, Nickstadt A, Bonas U (2009). Breaking the code of DNA binding specificity of TAL-type III effectors. Science.

[17] Moscou MJ, Bogdanove AJ (2009). A simple cipher governs DNA recognition by TAL effectors. Science.

[18] Deng D, Yan C, Pan X, Mahfouz M, Wang J, Zhu JK, Shi Y, Yan N (2012). Structural basis for sequence-specific recognition of DNA by TAL effectors. Science.

[19] Mak AN, Bradley P, Cernadas RA, Bogdanove AJ, Stoddard BL (2012). The crystal structure of TAL effector PthXo1 bound to its DNA target. Science.

[20] Zou J, Mali P, Huang X, Dowey SN, Cheng L (2011). Site-specific gene correction of a point mutation in human iPS cells derived from an adult patient with sickle cell disease. Blood.

[21] Sebastiano V, Maeder ML, Angstman JF, Haddad B, Khayter C, Yeo DT, Goodwin MJ, Hawkins JS, Ramirez CL, Batista LF (2011). In situ genetic correction of the sickle cell anemia mutation in human induced pluripotent stem cells using engineered zinc finger nucleases. Stem Cells.

[22] Eid J, Fehr A, Gray J, Luong K, Lyle J, Otto G, Peluso P, Rank D, Baybayan P, Bettman B (2009). Real-time DNA sequencing from single polymerase molecules. Science.

[23] Cermak T, Doyle EL, Christian M, Wang L, Zhang Y, Schmidt C, Baller JA, Somia NV, Bogdanove AJ, Voytas DF (2011). Efficient design and assembly of custom TALEN and other TAL effector-based constructs for DNA targeting. Nucleic Acids Res..

[24] Pruett-Miller SM, Connelly JP, Maeder ML, Joung JK, Porteus MH (2008). Comparison of zinc finger nucleases for use in gene targeting in mammalian cells. Mol. Ther..

[25] Warren L, Manos PD, Ahfeldt T, Loh YH, Li H, Lau F, Ebina W, Mandal PK, Smith ZD, Meissner A (2010). Highly efficient reprogramming to pluripotency and directed differentiation of human cells with synthetic modified mRNA. Cell Stem Cell.

[26] Guschin DY, Waite AJ, Katibah GE, Miller JC, Holmes MC, Rebar EJ (2010). A rapid and general assay for monitoring endogenous gene modification. Methods Mol. Biol..

[27] Maeder ML, Thibodeau-Beganny S, Osiak A, Wright DA, Anthony RM, Eichtinger M, Jiang T, Foley JE, Winfrey RJ, Townsend JA (2008). Rapid “open-source” engineering of customized zinc-finger nucleases for highly efficient gene modification. Mol. Cell.

[28] Fordis CM, Anagnou NP, Dean A, Nienhuis AW, Schechter AN (1984). A beta-globin gene, inactive in the K562 leukemic cell, functions normally in a heterologous expression system. Proc. Natl Acad. Sci. USA.

[29] Zein S, Li W, Ramakrishnan V, Lou TF, Sivanand S, Mackie A, Pace B (2010). Identification of fetal hemoglobin-inducing agents using the human leukemia KU812 cell line. Exp. Biol. Med. (Maywood).

[30] Banan M, Esmaeilzadeh-Gharehdaghi E, Nezami M, Deilami Z, Farashi S, Philipsen S, Esteghamat F, Pourfarzad F, Ali Imam AM, Najmabadi H (2012). CREB1 is required for hydroxyurea-mediated induction of gamma-globin expression in K562 cells. Clin. Exp. Pharmacol. Physiol..

[31] Erard F, Dean A, Schechter AN (1981). Inhibitors of cell division reversibly modify hemoglobin concentration in human erythroleukemia K562 cells. Blood.

[32] Christian M, Cermak T, Doyle EL, Schmidt C, Zhang F, Hummel A, Bogdanove AJ, Voytas DF (2010). Targeting DNA double-strand breaks with TAL effector nucleases. Genetics.

[33] Li T, Huang S, Jiang WZ, Wright D, Spalding MH, Weeks DP, Yang B (2010). TAL nucleases (TALNs): hybrid proteins composed of TAL effectors and FokI DNA-cleavage domain. Nucleic Acids Res..

[34] Wood AJ, Lo TW, Zeitler B, Pickle CS, Ralston EJ, Lee AH, Amora R, Miller JC, Leung E, Meng X (2011). Targeted genome editing across species using ZFNs and TALENs. Science.

[35] Huang P, Xiao A, Zhou M, Zhu Z, Lin S, Zhang B (2011). Heritable gene targeting in zebrafish using customized TALENs. Nat. Biotechnol..

[36] Sander JD, Cade L, Khayter C, Reyon D, Peterson RT, Joung JK, Yeh JR (2011). Targeted gene disruption in somatic zebrafish cells using engineered TALENs. Nat. Biotechnol..

[37] Tesson L, Usal C, Menoret S, Leung E, Niles BJ, Remy S, Santiago Y, Vincent AI, Meng X, Zhang L (2011). Knockout rats generated by embryo microinjection of TALENs. Nat. Biotechnol..

[38] Sun N, Liang J, Abil Z, Zhao H (2012). Optimized TAL effector nucleases (TALENs) for use in treatment of sickle cell disease. Mol. Biosyst..

[39] Sun N, Zhao H Seamless correction of the sickle cell disease mutation of the HBB gene in human induced pluripotent stem cells using TALENs. Biotechnol. Bioeng..

[40] Cradick TJ, Fine EJ, Antico CJ, Bao G (2013). CRISPR/Cas9 systems targeting beta-globin and CCR5 genes have substantial off-target activity. Nucleic Acids Res..

[41] Porteus MH (2006). Mammalian gene targeting with designed zinc finger nucleases. Mol. Ther..

[42] Miller AD, Bender MA, Harris EA, Kaleko M, Gelinas RE (1988). Design of retrovirus vectors for transfer and expression of the human beta-globin gene. J. Virol..

[43] Buchman AR, Berg P (1988). Comparison of intron-dependent and intron-independent gene expression. Mol. Cell Biol..

[44] Davis BM, Roth JC, Liu L, Xu-Welliver M, Pegg AE, Gerson SL (1999). Characterization of the P140K, PVP(138-140)MLK, and G156A O6-methylguanine-DNA methyltransferase mutants: implications for drug resistance gene therapy. Hum. Gene Ther..

[45] Neff T, Horn PA, Peterson LJ, Thomasson BM, Thompson J, Williams DA, Schmidt M, Georges GE, von Kalle C, Kiem HP (2003). Methylguanine methyltransferase-mediated in vivo selection and chemoprotection of allogeneic stem cells in a large-animal model. J. Clin. Invest..

[46] Bianchi N, Chiarabelli C, Borgatti M, Mischiati C, Fibach E, Gambari R (2001). Accumulation of gamma-globin mRNA and induction of erythroid differentiation after treatment of human leukaemic K562 cells with tallimustine. Br. J. Haematol..

[47] Cortesi R, Gui V, Gambari R, Nastruzzi C (1999). In vitro effect on human leukemic K562 cells of co-administration of liposome-associated retinoids and cytosine arabinoside (Ara-C). Am. J. Hematol..

[48] Fibach E, Kollia P, Schechter AN, Noguchi CT, Rodgers GP (1995). Hemin-induced acceleration of hemoglobin production in immature cultured erythroid cells: preferential enhancement of fetal hemoglobin. Blood.

[49] Haynes J, Baliga BS, Obiako B, Ofori-Acquah S, Pace B (2004). Zileuton induces hemoglobin F synthesis in erythroid progenitors: role of the L-arginine-nitric oxide signaling pathway. Blood.

[50] Keefer JR, Schneidereith TA, Mays A, Purvis SH, Dover GJ, Smith KD (2006). Role of cyclic nucleotides in fetal hemoglobin induction in cultured CD34+ cells. Exp. Hematol..

[51] Meiler SE, Wade M, Kutlar F, Yerigenahally SD, Xue Y, Moutouh-de Parseval LA, Corral LG, Swerdlow PS, Kutlar A (2011). Pomalidomide augments fetal hemoglobin production without the myelosuppressive effects of hydroxyurea in transgenic sickle cell mice. Blood.

[52] Mischiati C, Sereni A, Lampronti I, Bianchi N, Borgatti M, Prus E, Fibach E, Gambari R (2004). Rapamycin-mediated induction of gamma-globin mRNA accumulation in human erythroid cells. Br. J. Haematol..

[53] Moutouh-de Parseval LA, Verhelle D, Glezer E, Jensen-Pergakes K, Ferguson GD, Corral LG, Morris CL, Muller G, Brady H, Chan K (2008). Pomalidomide and lenalidomide regulate erythropoiesis and fetal hemoglobin production in human CD34+ cells. J. Clin. Invest..

[54] Osti F, Corradini FG, Hanau S, Matteuzzi M, Gambari R (1997). Human leukemia K562 cells: induction to erythroid differentiation by guanine, guanosine and guanine nucleotides. Haematologica.

[55] Witt O, Monkemeyer S, Ronndahl G, Erdlenbruch B, Reinhardt D, Kanbach K, Pekrun A (2003). Induction of fetal hemoglobin expression by the histone deacetylase inhibitor apicidin. Blood.

