# Supplementary material for: Nuclease-mediated gene editing by homologous recombination of the human globin locus
Source: Nucleic Acids Res. 2013 Oct 23;42(2):1365–78. doi: 10.1093/nar/gkt947 (PMC3902937; doi:10.1093/nar/gkt947)
Supplement: Supplementary Data [file supp_42_2_1365__index.html]

Nuclease-mediated gene editing by homologous recombination of the human globin locus — Nuclease-mediated gene editing by homologous recombination of the human globin locus — Supplementary Data 

# Nuclease-mediated gene editing by homologous recombination of the human globin locus

## Supplementary Data

files

**Files in this Data Supplement:**

- Supplementary Data - pdf file
