# Supplementary material for: Nuclease-mediated gene editing by homologous recombination of the human globin locus
Source: Nucleic Acids Res. 2013 Oct 23;42(2):1365–78. doi: 10.1093/nar/gkt947 (PMC3902937; doi:10.1093/nar/gkt947)
Supplement: Supplementary Data [file supp_gkt947_nar-02219-h-2013-File008.pdf]

```
βL1                caaacagacaccatg
βL2                caaacagacaccatggt
βL3                     agacaccatggtgcac
βL4                                 gcacctgactcctgt
βZFN R                                                                       ggggcaagg

             caacctcaaacagacaccatggtgcacctgactcctgaggagaagtctgccgttactgccctgtggggcaagg
             gttggagtttgtctgtggtaccacgtggactgaggactcctcttcagacggcaatgacgggacaccccgttcc

βR1                                               acctcttcagacggcaa
βR2                                                  tcttcagacggcaa
βR3                                                     tcagacggcaatgacg
βR4                                                               atgacgggacaccccgt
βZFN L                                                         gcaatgacg
```

Supplemental Figure 1. DNA binding sites of the β-globin TALENs and ZFNs. The ATG start site is highlighted in blue, the site of the sickle mutation is highlighted in red, and the nucleotides that are not conserved between β-globin and δ-globin are highlighted in yellow.

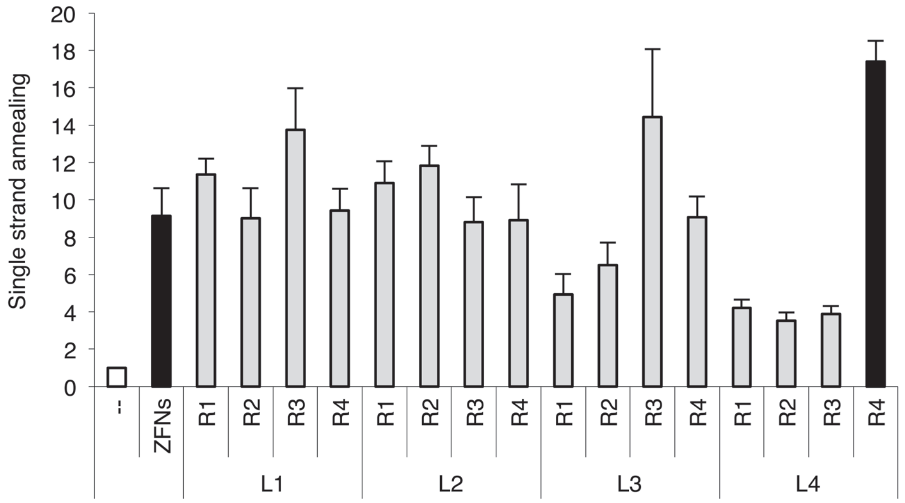

Supplemental Figure 2. Extrachromosomal single strand annealing (SSA) assay. HEK293T cells were co-transfected with a nuclease pair and a non-functional GFP gene that was disrupted by the insertion of the β-globin sequence that is recognized by the β-globin TALENs and ZFNs. Additionally, part of the GFP sequence on either side of the insertion is duplicated, such that when a break is induced, repair by single strand annealing generates a functional GFP gene. Black bars indicate the nuclease pairs that are used in the remainder of this study.

ZFN-L	ACG	GTA	GCA
Voit	RQQTLAQ	QRSSLVR	QTNTLGR
Sebastiano	RNITLVR	QRSSLVR	QPNTLTR

ZFN-R	AGG	GCA	GGG
Voit	RSDHLTN	QSTTLKR	RGDKLGP
Sebastiano	RGEHLRQ	QSGTLKR	RNDKLVP

Supplementary Figure 3. DNA recognition helices of the β-globin ZFNs. ZFNs used in this study and those recently described by Sebastiano *et al* [21] were independently designed to the same target sequence.

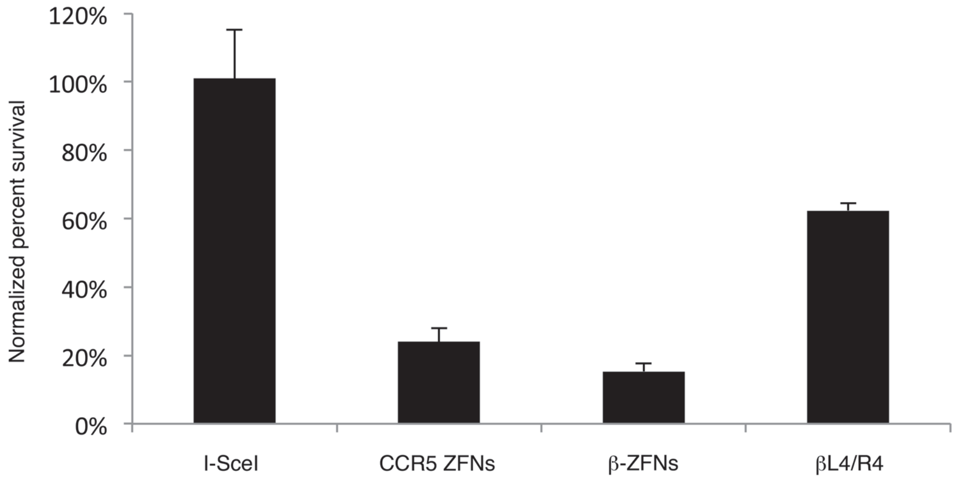

Supplemental Figure 4. Toxicity assay of β-globin nucleases. Toxicity of the β-globin ZFNs and TALENs was measured by co-trasnfection of the nuclease pairs with GFP into HEK293T cells. The ratio of GFP positive cells on day 6 to day 2 for each sample was normalized to samples transfected with the non-toxic nuclease I-SceI. CCR5 ZFNs[9] were used as an example of toxic nucleases.

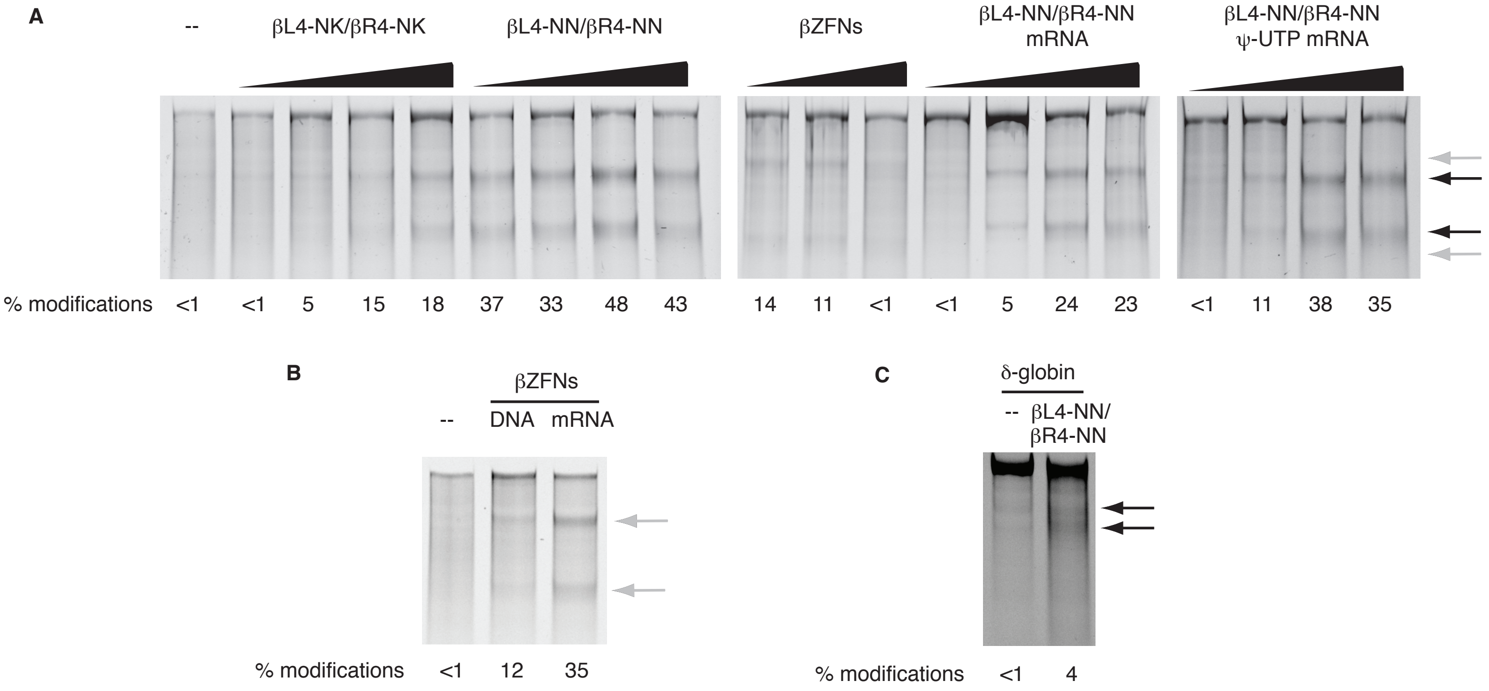

Supplemental Figure 5. β-globin TALEN and ZFN activity. (**A**) Titration from 0.1μg to 1.5μg of each -globin nuclease in the Surveyor nuclease assay. β-globin ZFNs were compared to L4/ R4 TALENs using the NK RVD, and L4/ R4 TALENs using the NN RVD delivered both as DNA, unmodified mRNA, and pseudo-UTP modified mRNA. (**B**) Comparison of the activity of β-globin ZFNs delivered as DNA or pseudo-UTP modified mRNA. (**C**) Activity of the best β-globin TALENs at the δ-globin locus. Black arrows indicate TALEN cleavage products, gray arrows indicate ZFN cleavage products.

```
γL1    5-gaggttatcaataagct-3
γL2          5-atcaataagctcct-3
γL3          5-atcaataagctcctagt-3

       ctgaggttatcaataagctcctagtccagacgccatgggtcatttcacagaggaggacaaggctactatcacaag
       gactccaatagttattcgaggatcaggtctgcgctacccagtaaagtgtctcctcctgttccgatgatagtgttc

γR2                                           3-taaagtgtctcctcc-5
γR3                                                3-tgtctcctcctgt-5
```

Supplemental Figure 6. DNA binding sites of the γ-globin TALENs. The ATG start site is highlighted in blue.

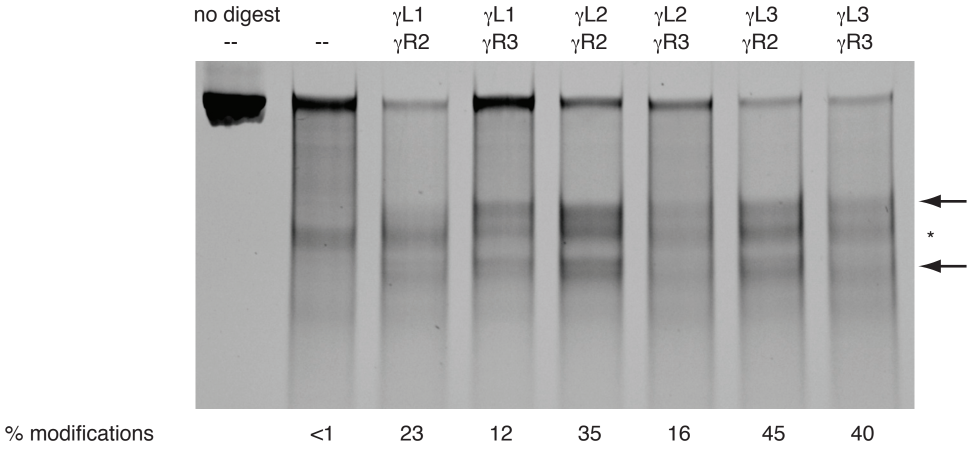

Supplemental Figure 7. Six pairs of active γ-globin TALENs. Activity of six pairs of γ-globin TALENs in the Surveyor nuclease assay. Arrows indicate specific cleavage products. * non-specific cleavage product.

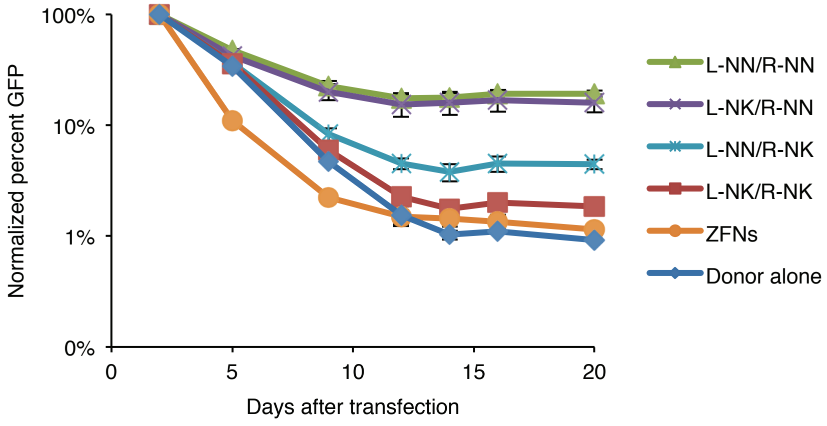

Supplemental Figure 8. Gene targeting using NN and NK β-globin TALENs. Comparison of all combinations of NN and NK left and right TALENs in the Ubc-GFP gene targeting assay.

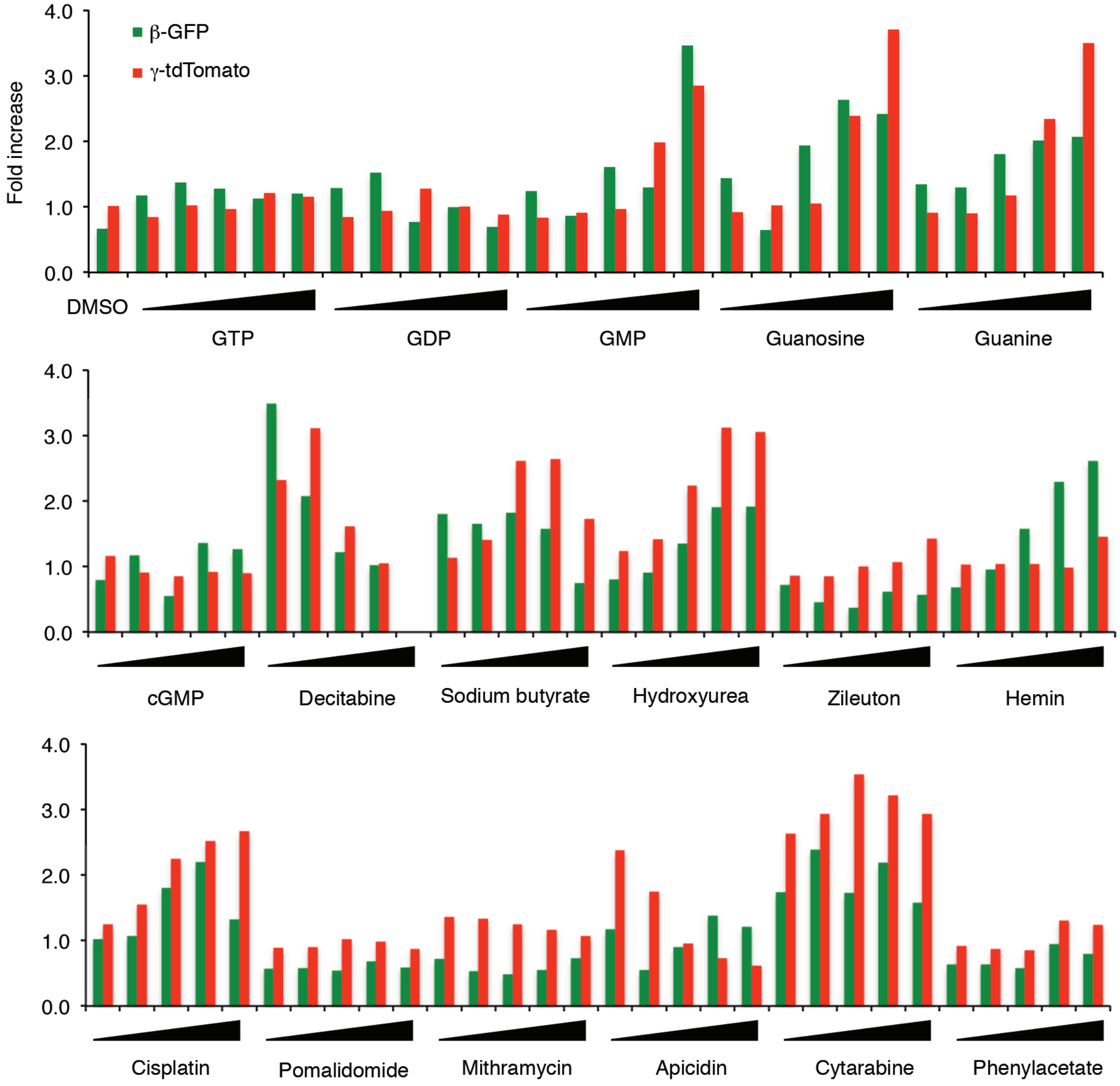

Supplemental Figure 9. Titration of globin-modulating compounds. Increase in GFP (green bars) and tdTomato (red bars) expression from the dual reporter cell line after treatment for four days with the indicated compound. The five drug concentrations used were 1x, 2x, 5x, 10x, and 20x and the 1x concentration for each is as follows: GTP, GDP, GMP, guanosine, guanine, cGMP, hydroxyurea, zileuton- 20μM; decitabine, cytarabine- 1μM; sodium butyrate- 60μM; hemin- 5μM; cisplatin- 400nM; pomalidomide- 2μM; mithramycin- 10μM; apicidin- 200nM; phenylacetate- 400μM.

β-globin targeting vector
5' arm
F: AGGCAGAAACAGTTAGATGTCC
R: TAGCAACCTCAAACAGACACCATG
3' arm
F: GGGGCAAGGTGAACGTGG
R: CTCTTTGCACCATTCTAAAGAAT
Site directed mutagenesis to introduce silent mutations (lower case) in β-globin cDNA
F: GCCCTcTGGGGaAAGGTcAACGTcGATGAgGTTGGcGGTGAaGCCCTcGGCAGGCTGCTGGTGGTCTACCC
R: CCATAGAGCCCACCGCATCC (sequence in BGH polyadenylation signal)
cDNA overlap PCR with ATG in 5' arm
F1: AGGCAGAAACAGTTAGATGTCC
R1: TTgACCTTtCCCCAgAGGGCgGTAACaGCAGAtTTCTCCTCAGGAGTCAGATGCACCAT
F2: GCCCTcTGGGGaAAGGTcAACGTcGATGAgGTTGGcGGTGAaGCCCTcGGCAGGCTGCTGGTGGTCTACCC
R2: CCATAGAGCCCACCGCATCC (sequence in BGH polyadenylation signal)
GFP overlap PCR with ATG in 5' arm
F1: AGGCAGAAACAGTTAGATGTCC
R1: TCCTCGCCCTTGCTCACCATGGTGTCTGTTTGAGGTTGCTA
F2: ATGGTGAGCAAGGGCGAGGA
R2: CCATAGAGCCCACCGCATCC (sequence in BGH polyadenylation signal)

γ-globin targeting vector
5'arm overlap with tdTomato
F1: TGTACACGCACATCTTATGTCT
R1: CCTCGCCCTTGCTCACCATGGCGTCTGGACTAGGAG
F2: ATGGTGAGCAAGGGCGAGG
R2: CCATAGAGCCCACCGCATCC (sequence in BGH polyadenylation signal)
3'arm
F: ATGGGTCATTTCACAGAGGA
R: GACTTTCAAAATCTACTCCAGC

Surveyor nuclease assay
β-globin
F: CCAACTCCTAAGCCAGTGCCAGAAGAG
R: CTCTTGGGTTTCTGATAGGCACTGACT
γ-globin
F: AAACGGTCCCTGGCTAAACT
R: TGAGAAGCGACCTGGACTTT
δ-globin
F: TCGACTGTTGCTTACACTTT
R: TAATCTGAGGGTAGGAAAAC

Unique tags for SMRT sequencing of cDNA targeting
F: CCAGACACTCTTGCAGATTAGTC
R: nnnTAGACCACCAGCAGCCT
nnn represents the following unique tags: AAA, TTT, ACT, TGA, AGC, TCG, ATC, TAG

Genomic PCR to confirm targeting
β-globin
F: CCTGCTGGCCGAAACTGA
R: ATGCAGAGATATTGCTATTGCCTTAAC
γ-globin
F: AGTGTGTGGACTATTAGTCAATAA
R: ATGAACTCTTTGATGACCTCC

qRT-PCR
β-globin
F: AACTGTGTTCACTAGCAACCTCAA
R: GAGTGGACAGATCCCCAAAGGA
γ-globin
F: ACTCGCTTCTGGAACGTCTGA
R: GTATCTGGAGGACAGGGCACT
GAPDH
F: GAAGGCTGGGGCTCATTT
R: CAGGAGGCATTGCTGATGAT

Supplemental Figure 10. List of primers used in this study
